# LONG-LIVED INDIVIDUALS PRESENTING WITH LARGE BREAST AND COLON TUMORS HAVE A LOWER RISK OF CONCURRENT METASTASIS

**DOI:** 10.1093/geroni/igz038.1722

**Published:** 2019-11-08

**Authors:** Benjamin Sweigart, Michelle R Johnson, Michael Voisine, Zeyuan Song, Kimberly Bertrand, Stacy L Andersen, Paola Sebastiani, Thomas T Perls

**Affiliations:** 1 Boston University, Department of Biostatistics, Boston, Massachusetts, United States; 2 Cambridge Health Alliance, Cambridge, Massachusetts, United States; 3 Boston University Medical Center, Boston, Massachusetts, United States; 4 Slone Epidemiology Center at Boston University, Boston, Massachusetts, United States; 5 Boston University School of Medicine, Boston, Massachusetts, United States

## Abstract

We hypothesized large tumors (stage T3 or T4) are less likely to metastasize in centenarians compared to younger patients. We analyzed 2004 to 2015 Surveillance, Epidemiology, and End Results (SEER) data for the most common cancer types (breast, colon, lung, and prostate) among patients with T3 or T4 tumors and compared rates of M1 (presence of metastases) at time of diagnosis according to ages 30-110 years. Among 44,066 breast cancer patients, metastasis rates fell after age 80 for T3 and after age 74 for T4 tumors. The relative risk of metastasis [RR] for T3 patients ages 90-110 years compared to ages 50-89 years was 0.73, 95% CI 0.57;0.94, and the RR for T4 patients was 0.48, 95% CI 0.42;0.55. Among 296,041 colon cancer patients, metastasis rates for T3 and T4 tumors steadily declined after age 60; RR for T3 patients was 0.66, 95% CI 0.62;0.71 and for T4 was 0.73, 95% CI 0.69;0.78 for the older and younger age groups. No difference in metastasis rates at diagnosis was observed for ages 90-110 with small cell and non-small cell lung cancers. Among 52,738 men presenting with stage T3 prostate cancer, the rate of metastasis steadily increased after age 70 (RR = 6.00, 95% CI 4.72;7.63) while there was no substantial difference in metastasis rate according to age for T4 patients. More work is needed to determine whether these findings are related to differences in screening and detection among those at older ages or whether they have a greater resilience to metastasis.

